# Analysis of velocity-mapped ion images from high-resolution crossed-beam scattering experiments: a tutorial review

**DOI:** 10.1140/epjti/s40485-015-0020-z

**Published:** 2015-07-28

**Authors:** Alexander von Zastrow, Jolijn Onvlee, David H. Parker, Sebastiaan Y.T. van de Meerakker

**Affiliations:** Radboud University, Institute for Molecules and Materials, Heijendaalseweg 135, Nijmegen, 6525 AJ Netherlands

**Keywords:** 34.50.-s, 37.10.Mn, Inelastic scattering, Differential cross sections, Molecular beams, Stark decelerator, Ion imaging, Image analysis, VMI

## Abstract

A Stark decelerator produces beams of molecules with high quantum state purity, and small spatial, temporal and velocity spreads. These tamed molecular beams are ideally suited for high-resolution crossed beam scattering experiments. When velocity map imaging is used, the Stark decelerator allows the measurement of scattering images with unprecedented radial sharpness and angular resolution. Differential cross sections must be extracted from these high-resolution images with extreme care, however. Common image analysis techniques that are used throughout in crossed beam experiments can result in systematic errors, in particular in the determination of collision energy, and the allocation of scattering angles to observed peaks in the angular scattering distribution. Using a high-resolution data set on inelastic collisions of velocity-controlled NO radicals with Ne atoms, we describe the challenges met by the high resolution, and present methods to mitigate or overcome them.

## Review

### Introduction

In crossed beam molecular scattering studies, the angular distribution of scattered products, which describes how the molecules are deflected during a collision, constitutes one of the most important of all observations. Precise measurements of this angular distribution directly probe the differential cross section (DCS) of the scattering process, providing unique and sensitive tests for the underlying potential energy surface(s) (PES).

In recent years, advanced experimental methods have been developed to record the angular scattering distribution efficiently and with high resolution. In particular the development of the velocity map imaging (VMI) [1] technique has significantly enhanced our ability to probe DCSs. With VMI, scattered molecules are detected state-selectively using laser-based resonance enhanced multiphoton ionization (REMPI) schemes. The ions are then imaged onto a two-dimensional plane such that their position on the detector reflects the recoil speed and direction of the scattered molecules, effectively resulting in a two-dimensional projection of the three-dimensional scattering Newton spheres.

Recently, we have pioneered a method to significantly enhance the resolution of the images using the Stark deceleration technique [2, 3]. The Stark decelerator is the equivalent for polar molecules of a linear accelerator for charged particles, and enables the production of packets of molecules with extremely narrow temporal, spatial and velocity spreads [4]. These tamed molecular beams enhance image resolution which is limited by reagent beam spreads in most other experiments. This was demonstrated by resolving quantum diffraction oscillations in the state-to-state DCSs for inelastic collisions between NO radicals and rare gas atoms [2, 3, 5].

Once the scattering image is measured, the differential cross section of the scattering process must be extracted from the image. This is done by first determining the angular intensity distribution from the outer rim of the image, which is then converted into a DCS. This procedure, however, is known to be a challenging task that is prone to error. The main challenges originate from three different classes of effects.

The first class contains various effects that cause a detection bias for certain post-collision velocities due to the kinematics of the experiment. These include Doppler and collision induced alignment effects, for instance, that result in a detection efficiency that depends on the angle between the post-collision velocity vector and the propagation or polarization direction of the laser. Another important and well-known member of this class is the so-called flux-to-density effect, that describes the detection probability of the scattered molecules related to the REMPI process. This probability is not equal for all scattered molecules, as REMPI probes the density of molecules at a given moment in time, whereas scattering cross sections are defined in terms of fluxes. As in a crossed beam experiment molecular beams typically overlap during a finite time, molecules that recoil with low laboratory velocities have a higher chance to be within the ionization volume when the probe laser is fired compared to molecules that have a large post-collision laboratory velocity.

The second class contains effects that relate to the inherent resolution in the images. In a crossed beam experiment, both reagent beams have nonzero velocity and angular spreads. The ensemble of colliding particles leads to the superposition of many Newton spheres, resulting in a blurring of the image. In general, however, this blurring affects every part of the image differently. On one side of the image, the different Newton spheres lie closer together than on the other side of the image. Therefore, both the measured intensity distribution and the inherent radial and angular image resolution will strongly depend on the scattering angle.

The third class contains effects that originate from the projection of the three-dimensional Newton spheres onto the two-dimensional detector plane. This ’crushing’ can lead to distortions and shifts of features that may be present in the angular scattering distributions such as rotational rainbows and diffraction oscillations. Again, these effects will depend on the kinematics of the experiment, and every part of the image will be affected differently.

In principle, all these effects occur simultaneously, are well understood, and can be accounted for. A proper analysis, however, requires detailed knowledge on the exact spatial, temporal and velocity distribution of the reagent beams, as well as on the spatial and intensity distribution of the ionization laser(s). Under typical experimental conditions, many of these parameters cannot precisely be measured and can only be estimated. Several strategies have been developed to analyze the images, and to extract DCSs from the images, focusing mostly on the effects within the first two classes discussed above [6–9].

In our experiments that employ a Stark decelerator in one of the two reagent beams, one of the beams has extremely narrow and well-calibrated temporal, spatial and velocity spreads. This results in a high inherent image resolution, in which structures such as rainbows and diffraction oscillations are resolved. The presence of these structures, as well as the sharp scattering rings that are typically recorded, leads to new challenges when the images are analyzed. On the other hand, the high resolution also offers the unique possibility to precisely study all effects listed above, and to investigate where one must be cautious when common analysis methods are used.

Here, we describe the challenges that can be encountered when analyzing high-resolution scattering images. We describe the source of systematic errors that can easily be made, and describe how the scattering information embedded in the images is compared best to theoretical predictions. This is illustrated using a high-resolution data set on inelastic state-to-state collisions between velocity-controlled NO radicals and Ne atoms, that were obtained using a crossed beam arrangement with 90 ° beam crossing angle.

We have chosen the NO-Ne system because the scattering of NO with rare gas atoms is one of the most intensely studied systems. Recently, Brouard and coworkers conducted a detailed study on rotational inelastic energy transfer using the NO-Ne system, and excellent agreement was obtained between experiment and theory [10]. Accurate theoretical predictions for differential cross sections exist, such that the differential cross sections that are derived from our high-resolution images can be directly compared to theoretical predictions. This facilitates the investigation of kinematic effects in the images that is the focus of this paper. In addition, the NO radical is very amenable to high-resolution scattering experiments using the combination of the Stark deceleration and velocity map imaging techniques [2, 3].

### Experiment

**Fig. 1:**
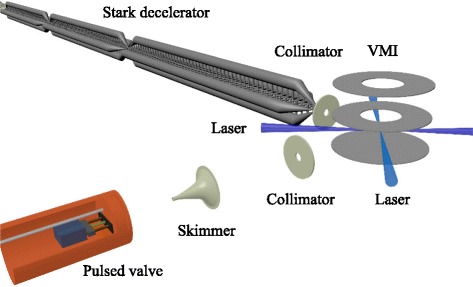
Schematic representation of the experimental set-up. A pulsed beam of NO radicals is passed through a 2.6-meter long Stark decelerator, and is scattered with a pulsed beam of rare gas atoms at a 90 ° beam intersection angle. The inelastically scattered NO radicals are state-selectively ionized without excess recoil energy using two pulsed lasers. The ions are subsequently detected using a standard velocity map imaging arrangement

**Fig. 2:**
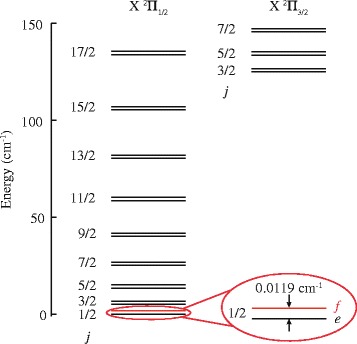
Rotational energy level diagram of NO (*X* ^2^ *Π*_*Ω*_,*v*=0) radicals. Two spin-orbit manifolds exist with *Ω*=1/2 and *Ω*=3/2. Each rotational level is labeled by the rotational quantum number *j*, and is split into two *Λ*-doublet components with *e* and *f* parity. The energy splitting between the *Λ*-doublet components of each rotational level is greatly exaggerated for clarity. The NO radicals that exit the Stark decelerator almost exclusively reside in the *j*=1/2*f* state

Measurements were performed in a crossed beam apparatus that is schematically shown in Fig. 1. The set-up, the Stark decelerator, and experimental procedures have been described in detail before [2–4]. Briefly, a molecular beam of NO radicals is formed by expanding a few percent NO in krypton through a Nijmegen Pulsed Valve [11]. After passage through a 3 mm diameter skimmer that is located about 100 mm from the nozzle orifice, the beam enters the 2.6-meter long Stark decelerator that consists of 317 pairs of high-voltage electrodes. The Stark decelerator is operated such that a packet of NO radicals emerges from the decelerator with a mean velocity of 370 m/s, a velocity spread of 2.4 m/s, and an angular spread of 0.1 ° (throughout this manuscript we refer to spreads as 1 *σ* of a Gaussian distribution). The Stark decelerator only transmits molecules that are in a low-field seeking quantum state, and approximately 99 % of the NO radicals that exit the decelerator reside in the upper *Λ*-doublet level of the *X* ^2^ *Π*_1/2_,*v*=0,*j*=1/2 rovibrational ground state. This state has *f* parity and is labeled hereafter as (1/2*f*); see Fig. 2 for a rotational energy level diagram of NO in its electronic and vibrational ground state.

At a distance of about 40 mm behind the decelerator the molecules pass a 3 mm diameter collimator. After an additional distance of 50 mm the NO radicals intersect a beam of rare gas atoms at a crossing angle of 90 °. The beam of rare gas atoms is produced from a commercially available pulsed valve (Jordan Inc., USA), that is placed at a distance of 110 mm from the beam crossing area. This beam is collimated by a 2 mm diameter skimmer and a 3 mm diameter collimator that are mounted 87 mm and 50 mm from the intersection point, respectively. The mean velocity of the Ne atoms was determined to be 912 m/s resulting in a collision energy of 485 cm ^−1^ (see section “Challenges in determining the center and radius of the scattering image” for a more quantitative discussion of the calibration of the collision energy). The atomic and molecular beams are synchronized such that the most intense parts of the beams arrive in the beam crossing area at the same time.

After the collisions, NO radicals are ionized state-selectively via a (1+1’) REMPI scheme, using two dye lasers that operate at wavelengths around 226 nm and 328 nm, respectively. The advantage of the (1+1’) REMPI scheme over the for NO more commonly used (1+1) REMPI scheme is the ability to ionize the NO radicals at threshold. This eliminates the blurring of the images due to ion recoil.

The NO ions are detected using VMI. The electric field geometry is produced by a repeller, extractor and grounded electrode, following the original design of Eppink and Parker [1]. The ions are accelerated towards a position sensitive detector that is placed at a distance of about 55 cm from the interaction region. The ion optics used are not optimized to allow a large ionization volume, i.e., the position at which ions are created has a significant influence on the imaging quality. To ensure a small ionization volume, both lasers are focused into the scattering volume. The first and second color lasers are attenuated to 3 *μ*J and 6 mJ, respectively, to prevent Coulomb repulsion effects from excessive signal levels and to prevent direct (1+1) REMPI by the first dye laser only. It is verified that all ionization signal disappears when blocking either of the two laser beams.

The detector consists of two microchannel plates in chevron configuration that are connected to a phosphor screen. Light pulses generated by impacting ions are recorded by a CCD camera (PCO Pixelfly 270XS, 1391 × 1023 pixels). The raw camera images are transferred to a PC and an event counting method is applied directly after acquisition. For every ion event with an intensity exceeding a previously set threshold, an area of 3×3 pixels is considered. In order to determine the mean ion impact coordinates *x*_m_ and *y*_m_, a Gaussian fit is performed on the projection of the event area to the *x* and *y* axes, respectively. These coordinates are stored in a measurement file for further analysis. Measurement results are displayed by binning the ion event coordinates into a two-dimensional histogram with adjustable resolution.

An automated background subtraction procedure is implemented in the experiment, which runs at a repetition rate of 10 Hz. Images for a given final product-state are first recorded by overlapping both the Ne and NO beams in time for 100 shots. Then, the Ne atom beam is delayed with respect to the NO packet, such that only background signal is recorded for 100 shots. This procedure is repeated in an alternating fashion and the scattering image can be inferred from the signal intensity difference of both images. A small fraction of NO radicals in the primary reagent beam resides in quantum states other than (1/2*f*) and causes unwanted signal, referred to as beam spot, in the forward direction when probing the various inelastic scattering channels. The background subtraction procedure overcompensates that beam spot and leads to a dip in the forward direction.

**Fig. 3:**
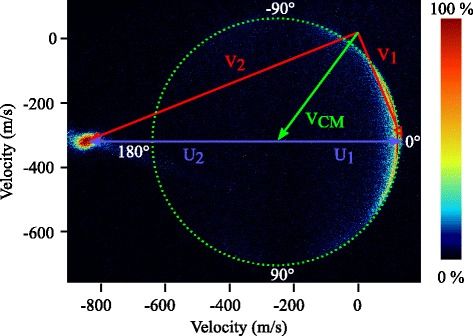
Velocity mapped ion image for the inelastic scattering process NO(*j*=1/2*f*)+Ne→NO(*j*=7/2*e*)+Ne. The Newton diagram pertaining to this scattering process is given as an overlay. The pre-collision laboratory (red) and center-of-mass (blue) velocity vectors of the NO and Ne beams, as well as the center-of-mass velocity vector (green) are indicated. Scattered molecules are expected on the so-called Newton circle indicated by the dashed green circle. Throughout this manuscript, scattering images are presented such that the mean relative velocity is oriented horizontally, and the laboratory zero-velocity is found in the top half of the image

Figure 3 shows the image obtained for scattering into the (7/2*e*) level as an example. The image intensity results from accumulating 100k shots of the experiment. The velocity vector (Newton) diagram is shown as an overlay. Red and blue arrows indicate the pre-collision velocity vectors of the NO and Ne beams in the laboratory and center-of-mass frames of reference, respectively. The green arrow indicates the center-of-mass velocity vector **V**_CM_. The image is presented such that the relative velocity vector is oriented horizontally. Due to conservation of energy and momentum, scattered NO radicals lie on a sphere with a radius determined by the collision energy, the reduced mass of the scattering partners, and the rotational energy that is taken up during the collision. This sphere is then projected onto the plane of the detector. The resulting Newton circle, that is centered around the center-of-mass velocity, is indicated by a green dashed circle. To quantify the scattering intensity along this circle, we use the convention that *θ*= 0 ° and *θ*= 180 ° correspond to forward and backward scattering, respectively. Furthermore, we use positive and negative values for *θ* to indicate scattering angles clockwise and anti-clockwise from *θ*= 0 °, respectively, i.e., the zero-laboratory velocity point is located at the top of the image for which *θ*<0.

Trace amounts of NO are entrained in the Ne beam, resulting in a relatively large beam spot on the left side of the image. Note that our background subtraction procedure does not compensate for this beam spot. The appearance of this beam spot is actually desired, as its position at the detector marks the velocity of the Ne beam. Together with the beam spot from the NO beam, the mean relative velocity vector of the colliding beams can be easily evaluated. As will be discussed in section “Challenges in determining the center and radius of the scattering image”, this is an important ingredient to appropriately calibrate the collision energy. For most final states, this beam spot is well separated from the region where scattered molecules appear on the detector. The beam spot will only partially overlap with the scattering signal for inelastic channels with relatively large backward scattered components, as typically found for excitation into states with *j*>7/2. In these cases, the scattering intensity in a small window around backscattered angles cannot be evaluated.

**Fig. 4:**
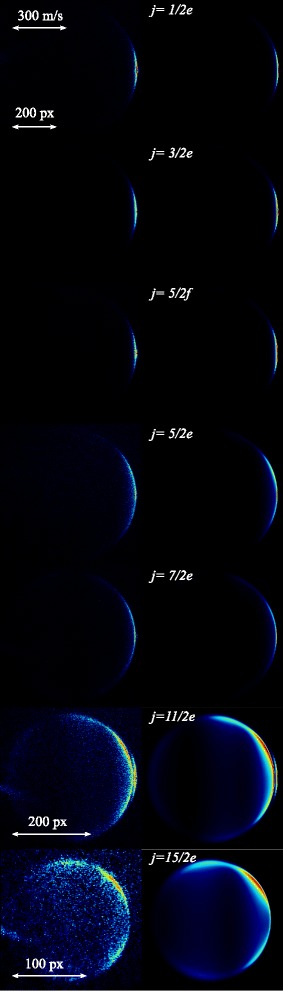
Scattering images for inelastic collisions of NO (j = 1/2f) radicals with Ne atoms. The left column shows the raw experimental scattering images for NO-Ne collisions, exciting the NO radicals (from top to bottom) to the (*j*=1/2*e*), (3/2*e*), (5/2*f*), (5/2*e*), (7/2*e*), (11/2*e*) and (15/2*e*) states. The images that result from full simulations of the experiment, using the differential cross sections from quantum scattering calculations based on *ab initio* potential energy surfaces as inputs, are shown in the right column. The image for the final state (11/2*e*) contains a second component near forward scattering, due to overlapping REMPI transitions (see also ref. [2])

### Results and discussion

#### Inelastic NO-Ne collisions

**Fig. 5:**
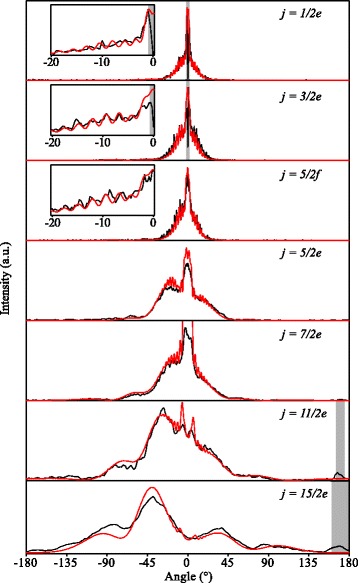
Angular scattering distributions. These distributions result from the experimental (black curves) and simulated (red curves) scattering images of Fig. 4, corresponding to the final states (from top to bottom) (*j*=1/2*e*), (3/2*e*), (5/2*f*), (5/2*e*), (7/2*e*), (11/2*e*) and (15/2*e*). The black and red curves are scaled with respect to each other. Parts of the distributions are shown on an enlarged scale in the insets to appreciate better the rapid diffraction oscillations that are recorded for inelastic channels with low rotational excitation

Figure 4 presents the experimental scattering images that are obtained for inelastic collisions de-exciting the NO radicals to the (1/2*e*) state (i.e., rotationally elastic collisions that induce a transition between the *Λ*-doublet components of the *j*=1/2 rotational ground state), as well as for collisions that excite the NO radicals into the (3/2*e*), (5/2*f*), (5/2*e*), (7/2*e*), (11/2*e*) and (15/2*e*) states. The evaluation of the angular scattering distributions from these experimental images will be explained in detail in the following sections. These distributions are shown in Fig. 5. We here briefly discuss the main features of the images.

The automated background subtraction procedure leads to an intensity dip at the location of the beam spot of the reagent NO packet. For final states with *j*<5/2, the dip overlaps with the scattering ring. Therefore the scattering intensity cannot be evaluated in this area and is marked with a gray box in the angular distributions. It is noted, however, that this area is very small due to the narrow velocity spread and high state purity of the reagent packet of NO. In fact, in most other crossed beam inelastic scattering experiments using NO, the measurement of excitation into final states with low *j* is not possible, as initial population of NO radicals in these states overshadows the scattering signal. The almost perfect state purity obtained here allows probes of these channels, including the exo-energetic (1/2*f*) → (1/2*e*) channel. For final states with *j*≥5/2, the beam spot of the reagent NO is well separated from the Newton circle, and the scattering intensity can be extracted from the image throughout the whole angular range.

The angular scattering distributions that are observed for the various inelastic scattering channels qualitatively follow the trend that is typically observed for inelastic collisions between atoms and diatomic molecules. For excitation with low *Δ* *j*, the scattering is dominated by glancing collisions with relatively high impact parameters leading to forward scattering. This is particularly prominent for the (1/2*e*), (3/2*e*) and (5/2*f*) channels where a nonzero scattering intensity is almost exclusively observed for angles close to *θ*= 0 °. As *Δ* *j* increases, lower impact parameters are required to excite the NO radicals, leading to a gradual change from forward scattering to side and backward scattering. This trend is clearly observed for the (5/2*e*), (7/2*e*), (11/2*e*), and (13/2*e*) channels. For channels with even higher *Δ* *j*, that are not probed in this work, scattering becomes predominantly backward scattered. Note that the (3/2*e*) and the (5/2*f*) channels display the same scattering intensity distribution, as these states belong to the same so-called parity pair, i.e., the DCS for scattering into a final state with rotational angular momentum *j* and parity *e* is identical to the DCS for scattering into a final state with rotational angular momentum *j*+1 and parity *f*, apart from a scaling factor close to unity [7, 12–14].

In addition to this general behavior, additional structure is observed in the scattering distribution. For low values of final *j*, rapid diffraction oscillations appear in the scattering distribution. For higher values of final *j*, broader structures such as rotational rainbows are found. In this manuscript, we focus primarily on the challenges that are present to analyze these high-resolution scattering images, and we will not further discuss the physical origins of the observed scattering distribution. For the latter, we refer the interested reader to the existing literature in which the rich dynamics of NO-rare gas collisions is discussed (see references [10, 15] and references herein).

#### Full simulations of the experiment

Full simulations of the experiment are performed using Monte Carlo trajectory simulations. The use of a Stark decelerator in the experiment brings the advantage that the spatial, velocity and temporal distributions of the NO packet are extremely well known from numerical simulations of the molecular trajectories inside the decelerator. These simulations therefore precisely yield the position, velocity and time for a given NO molecule that enters the interaction region. The parameters of the Ne atom beam, on the other hand, are less well known. This beam is assumed to have a Gaussian density distribution in the direction that corresponds to the molecular beam axis of the Stark decelerator, and a homogeneous distribution in the two other orthogonal directions, i.e., the NO radicals are assumed to scatter with a laminar gas flow. This approximation is valid under our experimental conditions, as the ionization volume is much smaller than the spatial extent of both beams, and as the interaction time of both beams is limited to < 20 *μ*s. For every NO radical that exits the Stark decelerator, the position and time at which this NO radical collides with a Ne atom is calculated from the spatial distribution of the Ne beam. The three velocity components of this Ne atom are then chosen randomly from Gaussian velocity distributions, where the longitudinal velocity and transverse angular spreads are assumed to be *Δ* *v*=2.5 *%* of the mean speed, and *Δ* *ϕ*=1 °, respectively.

For each NO-Ne pair, a Newton sphere is calculated in the center-of-mass frame from conservation of energy and momentum. NO radicals are distributed over the surface of this sphere according to a specific input DCS. Two types of DCSs are used in the simulations. Either hypothetical functions are used, or the DCSs of the scattering process that are obtained from quantum mechanical close-coupling (QM CC) calculations. In this work, we use QM CC calculations based on the PESs by Cybulski and Fernández [16]. The DCSs are typically sampled in steps of 0.1 °, and care was taken to distribute the molecules randomly over the sphere to prevent the occurrence of Moiré patterns and other artifacts. The final velocities of the scattered NO radicals in the laboratory frame were then calculated from a coordinate transformation between the center-of-mass and laboratory frames.

The positions of the scattered NO radicals at the moment of ionization are calculated by propagating all Newton spheres from the times at which the collisions occurred to the time at which the laser is fired. Only those molecules that are within the laser probe volume are counted. The laser ionization volume is not accurately known, and is modeled as a 2 mm diameter cylinder with a 2 mm length. The symmetry axis of the cylinder is chosen in the plane of the two incident beams, and rotated by 45 ° with respect to the incident NO beam (see also Fig. 1). Finally, a simulated image is constructed by binning the velocity components in the plane of the molecular beams in a two-dimensional velocity array.

The resulting simulated images, using the appropriate DCSs predicted by QM CC calculations as inputs, are shown in Fig. 4 as well. Calculations do not predict a significant difference for NO- ^20^Ne and NO- ^22^Ne collisions, such that the presence of ^22^Ne (natural abundance of about 10 %) is safely neglected.

#### Extracting the angular scattering distribution

To quantitatively compare experimental and simulated scattering images in Fig. 4, our first task is to extract the angular scattering distributions from the images by analyzing the scattering intensity in a small annulus around the rims of the images. As we will describe below, this is a surprisingly difficult task. Many effects related to kinematics of the crossed beam geometry affect the image (see section “Effects in the images”), severely complicating the analysis.

We start by converting the image intensity from cartesian grid coordinates to polar coordinates, and integrate the intensity within a small radial part from *r*_min_ to *r*_max_ in steps of *d* *r*=0.1pixels

1 $$ I(\theta)=\intop_{r_{\text{min}}}^{r_{\text{max}}}I(r,\theta)dr,  $$

where *I*(*r*,*θ*) is the intensity of the image in polar coordinates.

For given polar coordinates (*r*,*θ*) this conversion requires the determination of the scattering intensity at coordinates that are positioned somewhere within a single pixel. We interpolate between four neighboring pixels to deduce the intensity for an arbitrary position (*x*,*y*)

2 $$\begin{array}{@{}rcl@{}} I(x,y) & = & (1-\Delta x)\cdot(1-\Delta y)\cdot I(x_{i},y_{i})\\ &&+ \, (1-\Delta x)\cdot\Delta y\cdot I(x_{i},y_{i+1}) \\ &&+ \, \Delta x\cdot(1-\Delta y)\cdot I(x_{i+1},y_{i}) \\ &&+ \,\Delta x\cdot\Delta y\cdot I(x_{i+1},y_{i+1}), \end{array} $$

with *x*_*i*_<*x*<*x*_*i*+1_, *y*_*i*_<*y*<*y*_*i*+1_ being the *x* and *y* coordinates of the neighboring pixels, respectively, and *Δ* *x*=*x*−*x*_*i*_ and *Δ* *y*=*y*−*y*_*i*_.

#### Challenges in determining the center and radius of the scattering image

Evaluation of integral 1 requires the accurate determination of the center point of the image, i.e., the position at which we allocate *r*=0, and appropriate choices for the integrands *r*_min_ and *r*_max_. Both the center point and the integrands follow from the Newton diagram of the scattering process (see Fig. 3). Let **v**_NO_ and **v**_Ne_ be the mean pre-collision velocity vectors of the NO and Ne atom beams in the laboratory frame, respectively, i.e., these vectors denote the centers of the velocity distributions of the incoming molecular and atomic beams. The center point of the image is then given by the center-of-mass velocity vector

3 $$ \mathbf{V}_{\text{CM}}=\frac{m_{\text{NO}}\mathbf{v}_{\text{NO}}+m_{\text{Ne}}\mathbf{v}_{\text{Ne}}}{M},  $$

where *m*_NO_ and *m*_Ne_ are the masses of the NO molecule and Ne atom, respectively, and *M*=*m*_NO_+*m*_Ne_. The integrands *r*_min_ and *r*_max_ are chosen such that integral 1 is evaluated within a narrow annulus around the radius *r*_mean_ of the Newton circle, i.e., *r*_min_=*r*_mean_−*Δ* *r*_1_ and *r*_max_=*r*_mean_+*Δ* *r*_2_. The choices for *Δ* *r*_1_ and *Δ* *r*_2_ are in principle free, and can be made based on a compromise between desired signal-to-noise ratio and resolution of the resulting angular scattering distribution. In our experiments, the values for *Δ* *r*_1_ and *Δ* *r*_2_ typically range from one to four pixels. The radius *r*_mean_ of the Newton circle follows from conservation of energy and momentum, and is given by

4 $$ r_{\text{mean}} = |\mathbf{u}_{\text{NO}}| \sqrt{1-\frac{E_{\text{int}}}{E_{\text{coll}}}},  $$

where |**u**_NO_| is the length of the pre-collision velocity vector of NO in the center-of-mass frame (see also Fig. 3), *E*_coll_ is the collision energy, and *E*_int_ is the rotational energy difference between initial and final state of the inelastic scattering process.

We emphasize that for a meaningful analysis of high-resolution scattering images, it is absolutely essential to correctly determine the center-of-mass point and the mean radius of the Newton sphere. Herein lies also the most difficult challenge when analyzing the high-resolution images that are obtained using the Stark deceleration technique: in the parts of the images where the resolution is highest, the scattering intensity is distributed over a few pixels only. An error of only one or two pixels in the determination of **V**_CM_ and/or the radius *r*_mean_ will lead to large changes in the scattering intensity *I*(*θ*).

A commonly used and intuitive method in many crossed beam experiments is to derive the radius and center point from the scattering image itself. The underlying thought is that the scattering image resembles a circle, albeit blurred by the velocity spreads of both beams, from which a mean radius and center point can be determined. If one would connect the points in the image with highest intensity with each other, a circle that defines the Newton diagram of the scattering process will result, i.e., one would retrieve the unblurred image that would have been measured in the absence of any velocity spread or any other effect that reduces image resolution.

We have also implemented this approach, but we have found that the allocation of **V**_CM_ and *r*_mean_ by searching for the most intense parts of the image is fundamentally incorrect. The method can easily result in large systematic errors, that are unacceptably high to analyze the high-resolution images present in this work. The origin of this error is quantitatively described in the Appendix, and is related to both the kinematics of the crossed beam geometry experiment, and to the projection of the three-dimensional ion clouds on a two-dimensional detector plane.

We therefore refrain from using the image itself to determine the center-of-mass origin and the mean radius of the Newton sphere. Instead, we determine **V**_CM_ and *r*_mean_ in a two-step process. First, the center-of-mass point is evaluated from the measured beam spots of both reagent beams present in the experimental images (see Fig. 3). The relative velocity vector **v**_rel_ is found by connecting the center points of the beam spots, that were evaluated using Gaussian fits of both spots in the *x* and *y* directions. The center-of-mass point of the Newton diagram then follows from the known masses of the particles like in equation 3. The scattering angle *θ* is uniquely defined with respect to **v**_rel_, where *θ*= 0 ° points towards the beam spot of the parent NO beam. Note that the center-of-mass point is thus determined without knowledge of the location of the zero velocity point in the laboratory frame.

The second step is the determination of the radius *r*_mean_. For inelastic processes, the change in internal energy is known with spectroscopic accuracy. Referring back to equation 4, determination of the value for *r*_mean_ then requires a measurement of the velocity of the parent NO beam in the center-of-mass frame **u**_NO_, and the collision energy *E*_coll_ of the scattering process. Both the value for |**u**_NO_| and |**v**_rel_| (and thus *E*_coll_) are readily obtained from the analysis described above, provided that the calibration of the detector is accurately known, i.e., the conversion from pixel units to meter per second units must be accurately determined. For this, we exploit the extremely well calibrated velocities of the packets of molecules that exit the Stark decelerator, following a procedure as described in detail in ref. [3]. In this procedure, the Stark decelerator is programmed to produce packets of NO molecules with a mean velocity ranging between 350 m/s and 550 m/s and a very narrow velocity spread. The mean impact positions are determined for all beam spots, resulting in a linear relationship between impact position (in pixel units) and mean velocity of the NO packet (in meter per second units).

**Comparison between experimental and simulated scattering images** The QM CC calculations were performed with a scattering program for open-shell diatom-atom scattering, originally developed for collisions between OH molecules and rare gas (Rg) atoms [17]. We used the renormalized Numerov method for the propagation of the wave function on a grid from 4.5 to 45 bohr for NO-Ne. A basis set was used including all NO rotational levels up to *j*=20.5 and all partial wave contributions up to a total angular momentum of *J*=160.5 to reach convergence. State-to-state scattering cross sections at a collision energy of 485cm^−1^ were calculated using the *V*_sum_ and *V*_dif_ PESs by Cybulski and Fernández [16]. The obtained DCSs were used for the simulations described in section 1.

The angular scattering distributions that result from both the experimental and simulated images, where the radii and center points of the Newton diagrams are obtained as described above, are shown in Fig. 5. For all inelastic channels probed, excellent agreement between the experimental and simulated angular scattering distributions is obtained. All features, including broader structures with superimposed rapid diffraction oscillations, are well reproduced by the simulations. One can be, and perhaps should be, content with this agreement and conclude that the experiments are in full agreement with the QM CC calculations that are used as inputs to the simulations. However, one may perhaps also wish to compare experimentally determined DCSs with the DCSs predicted by theory. The curves shown in Fig. 5, however, do not yet represent the state-to-state DCSs of the scattering process, as both the experimental and simulated images contain all effects alluded to in the introduction, that are related to kinematics of the experiment.

The extraction of DCSs from images requires a quantitative discussion of all these effects. Some of these are obvious, such as the detection bias for low laboratory velocities which leads to strongly asymmetric scattering distributions around *θ*= 0 °. Others are more subtle and only become apparent after careful inspection of the images and scattering distributions. In the next section, we will discuss the most important effects that need to be taken into account when analyzing these images. We put particular emphasis on those effects that become particularly important when the images have high radial and angular resolution, as is the case when a Stark decelerator is used.

#### Effects in the images

**Doppler effect and collision induced alignment** The most straightforward effects in the images are those that originate from a mere velocity-dependent detection efficiency. One of the most well-known of these is the Doppler effect. Since the scattered molecules are distributed according to Newton spheres, a large range of post-collision laboratory velocities are present. Ideally, the detection laser ionizes all scattered molecules with equal efficiency, i.e., all Doppler-shifted absorption frequencies of the REMPI transitions are excited with equal probability. This can either be achieved by choosing a laser with sufficiently large bandwidth, by choosing sufficient laser power to broaden the transitions, by continuously scanning the laser frequency over the Doppler profile, or by choosing an optimal laser propagation direction that minimizes Doppler shifts. Like in most experiments, such free choices are not available in our experiments. The laser propagation direction is fixed in the plane of the beams, laser power must be chosen very low to prevent (1+1) REMPI transitions of NO, and spectral congestion in combination with the need to perform state-selective detection force us to use a dye laser with narrow bandwidth. In the experiments, the laser wavelength was fixed to the Doppler-free molecular absorption frequencies. This thus results in a detection bias for recoil velocities that is orthogonal to the laser propagation direction.

Another detection bias may arise from the so-called collision induced alignment. In the experiment, the direction of the final rotational angular momentum *j* ^′^ of the NO molecules can depend on the scattering angle *θ* [18, 19]. Therefore, the detection efficiency of scattered molecules depends on the angle between polarization direction of the probe laser and direction of the rotational angular momentum. Several studies on collision induced alignment in NO-Rg scattering have been performed in the past, and procedures to account for the effect, either based on full QM CC calculations [19] or on model calculations [7], are well-documented.

The consequences of both the Doppler and collision induced alignment effects to the image intensity distributions are relatively easily accounted for from the known geometry of the experiment. The Doppler effect requires the largest correction factors of up to a factor of three in image intensity for selected parts of the image. The influence of collision induced alignment was quantitatively investigated using the methodology as described by Brouard and coworkers [18], and correction factors in the 2 - 20 % range are found. In particular for final states with *j*<7/2, for which in our experiments the highest resolutions and signal-to-noise ratios are obtained, the collision products are rather forward scattered, and the correction factors are small. For higher values of *j* a larger correction is needed, but is in the majority of the images still small compared to the statistical noise of the experimental data.

**Image resolution and flux-to-density** Additional detection bias and complications originate from the kinematics of the experiment. We distinguish two main contributions here: (i) the non-uniform radial and angular resolution of the image due to the velocity spreads of both reagent beams, and (ii) the detection bias for certain post-collision velocities due to the temporal overlap of both beams and the finite size of the laser probe volume.

**Fig. 6:**
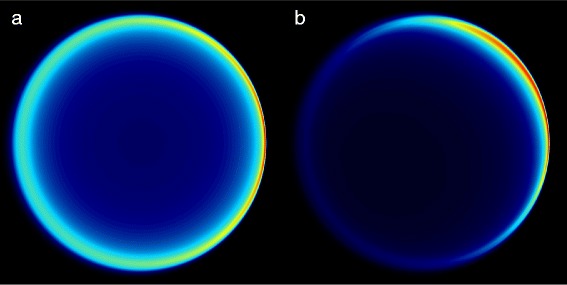
Simulated scattering images for elastic NO-Ne collisions. The images illustrate the asymmetry in intensity and resolution due to the velocity spreads of the beams alone (**a**) and due to a combination of beam spreads and the flux-to-density effect (**b**). The simulation parameters pertain to the experimental conditions; in (**a**) the finite laser probe volume is neglected, whereas in (**b**) the probe volume is taken into account. In both simulations an isotropic DCS is assumed

Let’s start with the inherent image resolution that results from the kinematics of the experiment. A collision between two particles can be represented by a Newton sphere, that is defined by its velocity radius and center-of-mass point. For such a Newton sphere, the scattering intensity is symmetric with respect to the relative velocity vector of the colliding particles. In a crossed beam experiment, however, collisions occur between particles from both beams, where each beam is characterized by its own angular and velocity spread. The effect of these spreads on the resolution of the image is illustrated in Fig. 6 (a). This image shows a simulation for elastic collisions between NO and Ne, assuming an isotropic DCS and an infinitely large detection volume, i.e., all scattered molecules are detected with equal efficiency. The parameters of the simulation are chosen such to represent the conditions as present in our experiment (see section “Full simulations of the experiment”), i.e., the NO radicals have a much smaller angular and velocity spread compared to the Ne atom beam.

From Fig. 6 (a) it is clear that the angular intensity distribution in the image is very inhomogeneous. In addition, the resolution is very different in the various parts of the image. Both effects stem from the superposition of many Newton spheres that originate from scattering events between particles from each beam. This is particularly apparent here, as the NO radicals have a very narrow velocity distribution. In the forward direction, the blurring of the image is governed by the velocity spread of the NO beam. Consequently, the Newton rings are found close together, and the resolution in this part of the image is high. In the backward direction, however, the resolution is mainly determined by the velocity distribution of the Ne atoms, and the Newton rings are spread over a larger area. For side scattering, an additional asymmetry can arise. In certain cases, the various Newton rings tend to overlap on one side of the relative velocity vector, while they occupy a larger area at the opposite side. A special case has been described by Chandler and coworkers to produce cold molecules with low translational temperature [20].

The situation is complicated further by the temporal overlap of the two reagent beams. In crossed beam experiments, the beams overlap with each other for some time before the detection laser is fired. During intermediate times, scattered molecules propagate in free flight according to their post-collision laboratory velocity vectors. Typically, the laser probe volume is small compared to the volume occupied by the scattered molecules. Fast molecules have a higher chance to fly outside the probe laser volume compared to slow molecules, leading to a detection bias for molecules with a low laboratory velocity vector. This effect is also often referred to as the flux-to-density effect, and has been described for crossed beam scattering experiments that focus on obtaining integral cross sections [21–24] as well as differential cross sections [6–9] before. In Fig. 6 (b) we illustrate using simulations how the images are affected by the flux-to-density effect. We use again simulation parameters as used to simulate Fig. 6 (a), i.e., we assume an isotropic DCS and reagent beams that pertain to our experimental conditions. In contrast to Fig. 6 (a) where a unit detection efficiency is assumed for all scattered particles, we now take the appropriate laser probe volume into account. Clearly, molecules that are side-scattered towards low laboratory velocities (*θ*∼−54 °) are detected most efficiently.

Figures 6 (a) and (b) show that the combination of kinematics and the flux-to-density effect results in asymmetric images, both in terms of image resolution and in terms of image intensity. The scattering signal is thus distributed differently over the image, even for an isotropic DCS, leading to strong gradients of image intensities. These intensity differences must be accounted for to extract a DCS from an experimental scattering image.

**Angular shift of features in the DCS** More subtle effects in the images arise when the DCS contains features that rapidly vary within a small angular interval, such as diffraction oscillations or (to a lesser extent) rainbows. Such features may appear in the image with a small but noticeable angular shift [2]. This shift is in part due to (i) the projection of Newton spheres onto the plane of the VMI detector, and (ii) the velocity spreads of both beams.

**Fig. 7:**
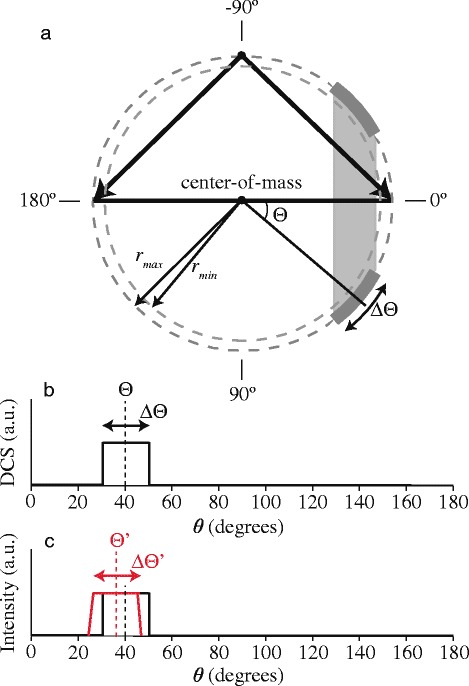
Simulation of the angular shift of small features in the DCS of a scattering process. **a** Schematic representation of the Newton diagram describing the scattering of two beams with particles of equal mass and equal pre-collision speed, with the relative velocity vector oriented horizontally. A DCS with a block feature as defined in panel (**b**) results in the gray area when the Newton sphere is crushed onto a two-dimensional plane. **c** Integration of the image intensity in an annulus between *r*_min_ and *r*_max_ results in an angular intensity distribution (shown by the red curve) that is shifted towards forward scattering with respect to the original DCS. This figure has been originally published in the supplement of reference [2] and was slightly adapted

The shift that is caused by the projection of Newton spheres onto the detector plane is qualitatively illustrated in Fig. 7. To model a diffraction feature in a DCS, consider a hypothetical DCS that exhibits a single block as schematically shown in panel (b) to Fig. 7. The block is centered at the angle *Θ* and has an angular extent *Δ* *Θ*. In panel (a) the Newton diagram is drawn that pertains to the “perfect” experiment in which two molecular beams, with zero angular and velocity spread, collide at 90 ° angle of incidence. For simplicity of arguments, we assume the two incoming particles to have equal mass and equal pre-collision speed. The block feature in the DCS is represented by the thick gray line segment on the Newton circle. The expected scattered intensity distribution that results from this hypothetical DCS is established by rotation around the relative velocity vector by the azimuthal angle *ϕ* from 0 ° to 360 ° (i.e. the angle *ϕ* specifies the direction within the plane that is perpendicular to the relative velocity vector). Hence, the block feature in the DCS transforms into a stripe in the image that is oriented perpendicular to the relative velocity vector. This stripe is schematically indicated in Fig. 7 (a) by the gray area. Since the angular intensity distribution in the images is analyzed by integrating the intensity within an annulus between *r*_min_ and *r*_max_ (see also section “Extracting a DCS from an image”), the block feature in the DCS appears shifted towards forward scattering (*θ*= 0 °), as is illustrated by the red curve in Fig. 7 (c).

**Fig. 8:**
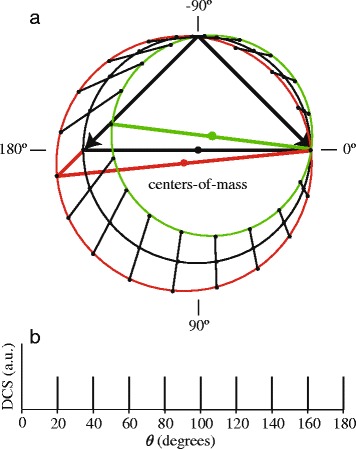
Distribution of angular features with a large velocity spread in one of the beams. **a** Schematic representation of the Newton diagram describing the scattering of two beams with particles of equal mass and equal pre-collision speed. One of the beams has zero velocity spread, whereas the other beam has a large velocity spread. Three Newton diagrams are drawn that correspond to scattering with the mean (black diagram) and two outermost values for the velocity (red and green diagrams). A DCS with a series of delta functions as defined in panel (**b**) results in line segments in the angular intensity distribution. The orientation of these line segments with respect to the mean relative velocity vector strongly depends on the scattering angle. This figure has been originally published in the supplement of reference [2] and was slightly adapted

Next we discuss the shift that appears due to the velocity spreads of the molecular beams, as is qualitatively illustrated in Fig. 8. Consider a hypothetical DCS that exhibits a series of delta-functions with an angular interval of 20 ° as schematically shown in Fig. 8 (b). This DCS models a series of equidistant diffraction peaks with infinitesimal width. Again, a Newton diagram is shown in panel (a) for two particles with equal mass and mean speed that collide at an angle of 90 °. No rotation over the azimuthal angle *ϕ* is used, but rather we investigate the influence of the velocity spread in the beams. Since the velocity spread of the atomic beam is much larger than the spread in the NO beam in our experiments, for the sake of simplicity of the arguments we assume all spreads for the NO beam to be zero. In panel (a) the three Newton diagrams are shown for the scattering with the mean velocity (black diagram), and both outermost values (red and green diagrams) within the velocity distribution of the atomic beam, respectively. For each Newton diagram, the position at which the delta-functions in the DCS will cause scattering intensity is indicated by a black point. Points that correspond to a single delta-function are connected by a straight line to guide the eye.

Clearly, the series of “diffraction oscillations” in the DCS appears as a series of line segments in the image. However, the orientation of these line segments strongly depends on the scattering angle *θ*: in some segments of the image the line segments are oriented radially towards the center of the circle, whereas in other segments of the image the line segments are oriented almost tangentially to the circle. Moreover, the structure is not symmetric with respect to the relative velocity vector. As before, when the image is analyzed by integrating the intensity within an annulus between *r*_min_ and *r*_max_, the oscillations appear shifted with respect to the angular positions of the original delta-functions. The same schematic figure and line of arguments can be made for the influence of the angular spread of the atomic beam, as well as the angular and velocity spreads of the NO beam, although the resulting line segments representing the diffraction oscillation peak positions are then oriented differently.

**Fig. 9:**
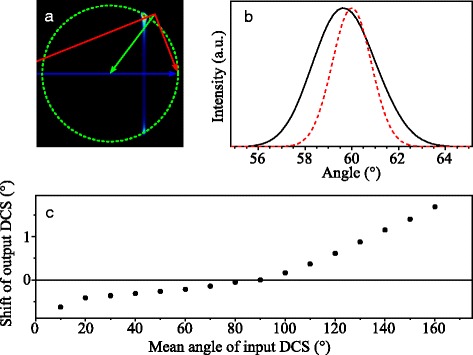
Angular shift of features in the DCS as present in the experiment. **a** Simulated scattering image that results from a hypothetical DCS that is centered around a mean position *Θ*= 60 °. **b** The hypothetical DCS (red dashed curve), together with the angular scattering intensity distribution (solid black curve) resulting from the simulated image. **c** Angular shift of the peak position of the angular scattering distribution with respect to the peak position of the input DCS, as a function of the mean position *Θ*

Figures 7 and 8 illustrate the physical origins of the shifts, but a more quantitative analysis is required to account for the shifts in our experiments. The shifts are investigated using a procedure as shown in Fig. 9. In this procedure, we simulate the ion images we expect to measure in the experiment, i.e., we use simulation parameters that pertain to NO-Ne collisions using our experimental conditions. Furthermore, we use model DCSs as input that are are characterized by a single Gaussian function, centered around a mean position *Θ* and with a width of *Δ* *Θ*= 2 ° (1*σ*). The model DCS for *Θ*= 60 ° is shown by the dashed red curve in Fig. 9 (b), and the corresponding simulated ion image is shown in Fig. 9 (a). The angular scattering distribution that results from the integrated intensity in the annulus at the rim of the image is shown by the black curve in panel (b). It is seen that the Gaussian function becomes asymmetric and its peak position is shifted by about 0.2 ° towards forward scattering.

The procedure is repeated for several Gaussian functions with mean positions *Θ* ranging from *Θ*=0 ° to 180 °. The resulting shifts as a function of *Θ* are shown in Fig. 9 (c). It is seen that the shifts become larger for values of *Θ* towards 0 ° and 180 ° and vanish for *Θ*∼ 90 °.

The shifts and distortions as discussed and analyzed above have a number of important consequences for high-resolution imaging experiments: (i) Experimentally determined structures in angular intensity distributions in high-resolution experiments do not directly reflect structures in the DCS. A noticeable shift can occur which, if not properly taken into account, leads to an erroneous allocation of the scattering angle *θ* to the observed structures. (ii) This shift is not symmetric with respect to the forward scattering angle *θ*= 0 °. In contrast to an intuitive picture on the use of VMI in crossed beam scattering, the peak positions for features in the DCS do not appear in the image symmetrically around the relative velocity vector before collision. It is emphasized that this is even the case for the hypothetical situation in which ultimate time-slicing can be achieved and in which only the equators of the Newton spheres are imaged. The shifts and asymmetry already result from pure kinematic effects related to beam spreads.

#### Extracting a DCS from an image

In the previous sections we have discussed a variety of effects that can affect the scattering distribution in the experimental images, and we have shown that appropriate simulations of the experiment can fully account for these effects. Almost perfect agreement between experimental and simulated images and angular scattering distributions is obtained (see again Figs. 4 and 5). We now return to the inverse question: for a given measured scattering image, how can one extract a DCS that can directly be compared to a DCS predicted by theory?

We will distinguish two different types of DCSs that can occur. In the first, the DCS contains relatively broad structures such as rotational or l-type rainbows. These features are typically more than 10 ° wide, and separated by a relatively large angular interval. The scattering images from Fig. 4 pertaining to the final states (5/2*e*), (7/2*e*), (11/2*e*) and (15/2*e*) belong to this category. In the second, the DCS contains very rapid oscillatory structures such as diffraction oscillations. The width and spacing of individual features may be on the order of only one degree, such as present for the final states (1/2*e*), (3/2*e*) and (5/2*f*). Both types of DCS require a different treatment of the scattering image.

**Fig. 10:**
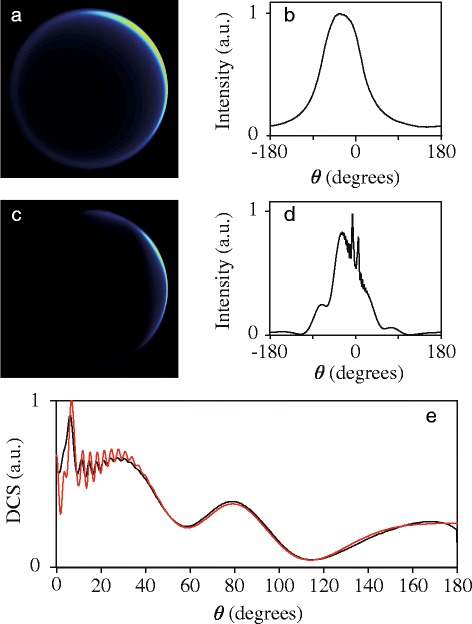
Illustration of the DCS extraction process using the (11/2*e*) final state as an example. **a** Simulated scattering image, using an isotropic DCS as input. **b** Angular scattering distribution that results from the image in panel (**a**), which is referred to as the apparatus function. **c** Simulated scattering image, using the DCS for the (11/2*e*) final state from quantum scattering calculations as input. **d** Angular scattering distribution that results from the image in panel (**c**). **e** Extracted DCS (black curve) that is obtained using the apparatus function to correct the angular scattering distribution, together with the DCS from quantum scattering calculations (red curve)

**DCS contains relatively broad structures such as rainbows** For structured DCSs that contain relatively broad features such as rainbows, extraction of a DCS from the image is relatively straightforward. Once the correct center point and radius of the Newton diagram is found following the procedures outlined in section “Challenges in determining the center and radius of the scattering image”, DCS extraction procedures as used and described by others can be used [6–9]. We follow the procedure described by Chandler and coworkers [6], which is schematically illustrated in Fig. 10. The method starts by evaluating an apparatus function, i.e., one simulates the scattering image using an isotropic DCS as input. These simulations are based on a best-guess of the beam parameters and kinematics of the experiment. An example of such an image pertaining to our experimental conditions (using the (11/2*e*) final state as an example) is shown in Fig. 10 (a). The angular distribution that is derived from this image, shown in panel (b), is referred to as the apparatus function. This curve carries the information on how effective certain collision angles are registered by the experiment and image analysis methods, and can be used to correct the scattering intensity distribution that is derived from a given experimental scattering image.

This is illustrated in the lower half of Fig. 10, that schematically shows the procedure for the (11/2*e*) final state as an example. The simulated scattering image for this channel, that is based on the DCS predicted by quantum scattering calculations, is shown in panel (c). The angular scattering distribution that results from this image is shown in panel (d). The scattering distribution is clearly asymmetric, with in general much larger intensity for angles *θ*< 0 ° compared to the corresponding angles with *θ*>0 °. The DCS of the scattering process is then extracted by dividing this scattering distribution by the apparatus function, as is shown in panel (*e*). The resulting angular distribution is fully symmetric with respect to *θ*= 0 °; the extracted DCS (black curve in panel (*e*)) is then simply defined by the scattering distribution within the interval 0 ° ≤*θ*≤ 180 °. It is seen that the procedure retrieves the *ab initio* DCS (red curve), blurred by the experimental resolution, in which the broad features and structures are found at the correct scattering angles and with the correct relative scattering intensity.

This procedure only leads to correct results, and the simulations only fully reproduce the experimental scattering images, if the parameters used in the simulations exactly match the experimental conditions. In case they don’t, Chandler and coworkers described an iterative procedure to evaluate the DCS by repeating the process of comparing experimental and simulated images [6]. In each iteration, the DCS that is found is corrected based on the results of the previous iteration. This process is continued until the experimental and simulated images have best agreement. This iterative procedure thus extracts the most likely DCS from the experimental image, although information of experimental conditions is incomplete.

We have found that for our conditions, the first step in this iterative process already results in a very good agreement between simulated and experimental images. Further iterations do not yield better agreement within the noise of the experimental data. We thus conclude that the beam parameters and general kinematics of the experiment assumed in the simulations closely resemble the actual conditions present in the experiment. It is noted that this situation is accomplished in part by a very careful calibration of experimental conditions, and in part by the extremely well known and calibrated phase-space distribution of the packet of NO radicals exiting the Stark decelerator.

**Fig. 11:**
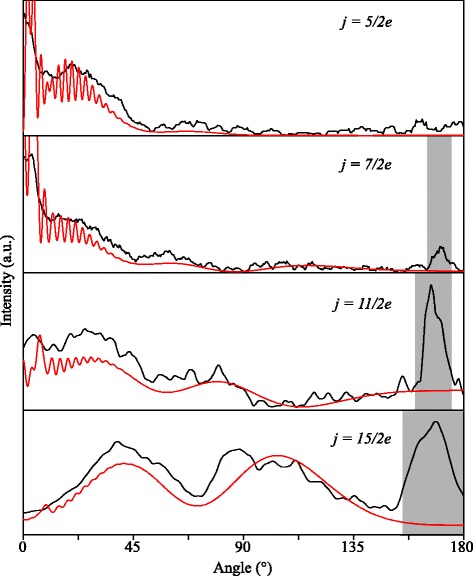
Experimentally determined differential cross sections. The experimentally determined and corrected DCSs (black curves) are shown together with the cross sections resulting from quantum scattering calculations (red curves), for the final states (from top to bottom) (5/2*e*), (7/2*e*), (11/2*e*) and (15/2*e*)

The DCSs that are extracted from the experimental scattering images for the final states (5/2*e*), (7/2*e*), (11/2*e*) and (15/2*e*) are shown in Fig. 11, together with the predictions that result from the *ab initio* quantum scattering calculations. It is seen that excellent agreement with the predictions is obtained. As described above, the presence of the secondary beam spot shows up in the DCS graphs at around 170 ° and the DCS cannot be evaluated there. These areas are therefore marked with a gray box.

**DCS contains rapid oscillatory structures such as diffraction oscillations** For DCSs that contain rapidly varying structures such as diffraction oscillations, the forward iterative methods described above can in principle also be used to extract a DCS from the experimental image: the extracted DCS containing all diffraction peaks is iterated until the simulated image exactly resembles the measured image. However, in this case, to reach convergence in the iterations is rather complicated. As described in detail in section “Angular shift of features in the DCS”, diffraction peaks in the experimental images appear shifted with respect to the underlying DCS. Considering the signal-to-noise ratio with which these peaks are measured, and the very small angular spacing between adjacent peaks, it is very challenging to perform extensive iterative analysis procedures.

Instead, for the channels where the DCS is dominated by diffraction structures, we feel the best and most honest method to compare experiment with theory is to directly compare the angular scattering distributions that are derived from the raw experimental and simulated images, such as presented in Fig. 4. Although in both distributions the positions at which peaks are found are shifted with respect to the *ab initio* DCS, we trust that theory adequately describes the experiment if good agreement between both curves is found, i.e., we refrain from trying to directly extract a DCS from the experimental image.

It is noted that we have recently developed an alternative method to measure diffraction oscillations. This method exploits the benefits of a counterpropagating beam geometry, in which the velocity spreads of the reagent beams on the angular resolution of the images is minimized [5]. In addition, the counterpropagating geometry results in images that are symmetric with respect to the relative velocity vector. This allows the use of inverse Abel transformation methods that enhance the resolution further. The Abel transformation also compensates for the angular shift of peak positions in the raw experimental images, such that extracted DCSs can directly be compared to the output of quantum scattering calculations. We therefore feel that the counterpropagating geometry is the preferred method to experimentally study diffraction oscillations. If a counterpropagating geometry is experimentally not available, as is the case in the work presented here, the preferred method is to directly compare the raw experimental image with accurate simulations based on the theoretically predicted DCS.

## Conclusions

The combination of the Stark deceleration with the velocity map imaging technique allows the measurement of high-resolution scattering images in crossed beam scattering experiments. The resolution that is obtainable is sufficient to record, for instance, diffraction oscillations or rainbow features in state-to-state inelastic cross sections. This was illustrated here for inelastic scattering of NO (*X* ^2^ *Π*_1/2_,*j*=1/2*f*) radicals with Ne atoms, for which a total of seven inelastic channels were probed. Excellent agreement is obtained with the cross sections that result from quantum scattering calculations that are based on recent *ab initio* potential energy surfaces.

To extract differential cross sections from the scattering images, extreme care must be taken. We have shown that many effects can be present in the images that can lead – when not appropriately taken into account – to severe misinterpretation of the data. We have given a comprehensive and coherent overview of all artifacts that may occur, ranging from angular shifts with which features in a DCS emerge in a scattering image to the more well-known flux-to-density and other kinematic effects.

All effects and artifacts discussed here are generally present in all crossed beam scattering experiments that employ velocity map imaging as a detection technique. However, in experiments where exclusively conventional molecular beams are used, the resolution of the experiment is such that the most delicate effects are safely neglected. In contrast, the high image resolution afforded by the Stark decelerator makes the experiment extremely sensitive to all effects present in the images, even the very subtle ones.

We have described that in particular major errors in the data analysis can result when the Newton circle governing the scattering kinematics is not correctly determined. Simply determining the relevant parameters by fitting a circle to an annulus of highest intensity in the scattering image, a common strategy in crossed beam experiments that employ VMI, should be carefully considered. We have shown that for high-resolution images, where the rims of the images are typically only a few pixels wide, it is very difficult to extract the Newton diagram from the image itself, potentially leading to large systematic errors when the angular scattering intensity is evaluated.

In this manuscript we describe an alternative and more accurate method that is appropriate to analyze the high-resolution images. The method optimally exploits the well-calibrated packets of molecules that exit the Stark decelerator. By recording beam spots on the detector as a function of the programmable final velocity of the packet, the detector is accurately calibrated. This calibration relates pixel units to meter per second units within a large area of the detector, facilitating the meaningful interpretation of velocities that are less well known in the experiment, such as the mean beam speed of the scattering partner. Together, this yields an accurate determination of the kinematics present in the experiment, without relying on the scattering image itself. After establishing the kinematics, common analysis methods can be used to extract the differential cross section from the scattering image.

Although the calibration method is relatively easy and elegant, one should run the time-consuming calibration measurements typically several times a day to compensate for drifts in voltages and charging up effects. However, the message is clear; to extract meaningful information from scattering images that have extremely high resolution, an often quoted phrase in the crossed beam scattering community applies: "one needs to spend just as much effort in careful calibration of the experiment as in recording the collision data itself".

## Appendix

To analyze scattering images in a crossed beam scattering experiment, the center-of-mass point **V**_CM_ and the radius *r*_mean_ of the Newton circle must be known, i.e., the parameters that define the Newton circle for a collision between two hypothetical particles, where each particle has a velocity corresponding to the mean speed of the beam distribution. In most crossed beam experiments, however, the mean velocities of the reagent molecular beams are not precisely known, hampering the precise measurement of the collision energy *E*_coll_ and the center point defined by **V**_CM_.

A commonly used and intuitive method in many crossed beam experiments is to derive these parameters from the scattering image itself. We have also followed this approach, and employed a variant of the Hough transformation [25] to determine the center and radius of the circle. The method uses three parameters that uniquely define a circle, which are the center point *x*_c_, *y*_c_ and radius *r*. The algorithm then transforms image data *I*(*x*,*y*) to the Hough space *H*:

5 $$ I(x,y)\rightarrow H(x_{\mathrm{c}},y_{\mathrm{c}},r)  $$

where *x* and *y* are the pixel coordinates in the initial image. The transformation function runs over all possible values of (*x*_c_,*y*_c_,*r*) and assigns an intensity to it which is the integrated intensity found on that circle. The integration function uses a version of the Bresenham algorithm which was modified to integrate circles [26]. The circle that is allocated the highest intensity defines **V**_CM_ and *r*_mean_.

The allocation of **V**_CM_ and *r*_mean_ by searching for the most intense parts of the image, however, is fundamentally incorrect. This is related to the kinematics of the experiment, and to the projection of the three-dimensional ion clouds on a two-dimensional detector plane. As discussed in more detail in section 1, the velocity spreads in the reagent beams lead to blurring of the image, but this blurring is in principle *not* symmetric with respect to the Newton circle of the scattering process. In parts of the image, the beam spreads will lead to a maximum scattering intensity at values for *r* that are different from *r*_mean_. Thus, searching for a radius where the scattering intensity is maximal will lead to a misinterpretation of the Newton circle.

**Fig. 12:**
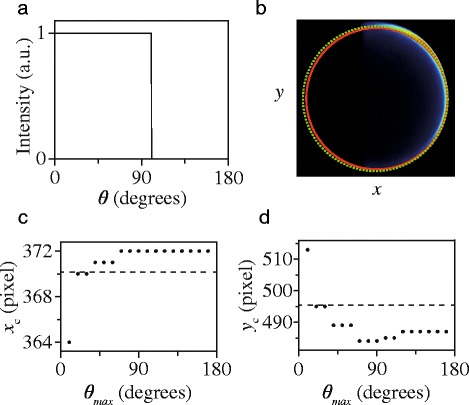
Illustration of misinterpretation of center and radius of Newton circle. The figure shows the error when these parameters are derived from a scattering image. **a** Hypothetical block DCS that is unity from *θ*= 0 ° to *θ*_*max*_= 100 ° and zero otherwise. **b** Scattering image that results from this block DCS, together with the true Newton diagram for this scattering process (green) and the Newton ring that is found by fitting the scattering intensity of the image using the Hough transformation (red curve). (c and d) Center point coordinates (*x*_c_ and *y*_c_ in panel (**c**) and (**d**), respectively) of the Newton diagram resulting from the Hough transform for hypothetical block DCSs as a function of the value for *θ*_max_. The center points of the true Newton diagram are indicated by horizontal dashed lines

The error, however, is relatively small when both reagent beams have a large and comparable velocity spread, a situation that is found in a typical crossed beam scattering experiment. If, however, one of the beams has a much larger spread than the other, as is the case in our experiments, the error becomes unacceptably large. This is illustrated in Fig. 12, where we present a method to quantitatively investigate the error that is made when using the algorithm to find **V**_CM_ and *r*_mean_. We performed full simulations of the experiment, using beam parameters that pertain to our experimental conditions (see section 1), using hypothetical block DCSs as input. These block DCSs are constant from *θ*=0 ° up to a given final value *θ*_max_; the input DCS for *θ*_max_=100 ° is shown in Fig. 12 (a) as an example. A scattering image is simulated, and subsequently analyzed using the algorithm described above. The results for *θ*_max_=100 ° are shown in Fig. 12 (b), where the simulated image is shown with superimposed the Newton circle that is calculated from *v*_NO_ and *v*_Ne_ that are used as input to the simulation. The location of the Newton ring that is defined by **V**_CM_ and *r*_mean_ is shown by the green dashed circle. Also shown is the Newton ring (red circle) that is found by using the algorithm.

Clearly, the red and dashed green circles do not overlap; both the center point and the radius of the circle are not correctly evaluated by the algorithm. Several of these simulations were performed for block DCSs with values for *θ*_max_ ranging from 10 ° to 180 °. The simulated images are analyzed using the algorithm, and the center point (*x*_*c*_,*y*_*c*_) of the Newton circle is compared to the true center point **V**_CM_ of the scattering process. The results are shown in Fig. 12 (c) and (d) for *x*_*c*_ and *y*_*c*_, respectively, as a function of the block length of the hypothetical DCS. The horizontal dashed lines indicate the center point coordinates of **V**_CM_.

It is seen that for small values of *θ*_max_, the algorithm makes a large error. This is in part due to the fact that the scattering intensity is spread over a very small area compared to the size of the Newton circle. It is therefore difficult to fit a circle to this small segment. For larger block lengths, however, there is still a significant error of up to a few pixels. For high-resolution scattering images, where the width of the ring is only a few pixels wide, such error is unacceptable, and the method must be discarded to analyze our images.
